# Reduction of delayed onset muscle soreness by a novel curcumin delivery system (Meriva®): a randomised, placebo-controlled trial

**DOI:** 10.1186/1550-2783-11-31

**Published:** 2014-06-18

**Authors:** Franchek Drobnic, Joan Riera, Giovanni Appendino, Stefano Togni, Federico Franceschi, Xavier Valle, Antoni Pons, Josep Tur

**Affiliations:** 1 Olympic Training Centre (CAR), Barcelona, Spain; 2 Università degli Studi del Piemonte Orientale, Novara, Italy; 3 Indena s.p.a., Milano, Italy; 4 University of Barcelona, School of Sports Medicine, Medical Services, FCBarcelona, Spain; 5 Department Biologia Fonamental i de la Salut, University of Illes Balears, Mallorca, Spain

**Keywords:** Meriva®, Curcumin, DOMS, Sport nutrition

## Abstract

**Background:**

Delayed onset muscle soreness (DOMS) due to eccentric muscle activity is associated with inflammatory responses and production of reactive oxygen species (ROS) that sustain both inflammation and oxidative stress. Curcumin, a powerful promoter of anti-oxidant response, is one of the best-investigated natural products, and is now commercially available as a lecithin delivery system (Meriva®, Indena SpA, Milan) with improved bio-availability. The aim of this study was to test whether curcumin could attenuate damage from oxidative stress and inflammation related to acute muscle injury induced by eccentric continuous exercise

**Methods:**

This was a randomised, placebo-controlled, single-blind pilot trial. Twenty male healthy, moderately active volunteers were randomised to curcumin given as the Phytosome® delivery system 1 g twice daily (200 mg curcumin b.i.d.) or matching placebo. Supplementation was initiated 48 hours prior to a downhill running test and was continued for 24 hours after the test (4 days in total). Muscle damage was quantified by magnetic resonance imaging, laboratory tests and histological analyses on muscle samples obtained 48 hours after the test. Patient-reported pain intensity was also recorded.

**Results:**

Subjects in the curcumin group reported less pain in the lower limb as compared with subjects in the placebo group, although significant differences were observed only for the right and left anterior thighs. Significantly fewer subjects in the curcumin group had MRI evidence of muscle injury in the posterior or medial compartment of both thighs. Increases in markers of muscle damage and inflammation tended to be lower in the curcumin group, but significant differences were only observed for interleukin-8 at 2 h after exercise. No differences in markers of oxidative stress and muscle histology were observed

**Conclusions:**

Curcumin has the potential for preventing DOMS, as suggested by its effects on pain intensity and muscle injury. Larger studies are needed to confirm these results and further clarify the mechanism of action of curcumin.

## Background

Delayed onset muscle soreness (DOMS) is a combination of muscle pain and stiffness occurring several hours after unaccostumed exercise, particularly when eccentric muscle activity is involved [1]. Both physically inactive individuals and athletes are familiar with DOMS, which may limit physical function for several days after exercise [2]. Over the past two decades, a large number of studies have been conducted to test different strategies for preventing DOMS [3-7], but no specific single intervention has been conclusively demonstrated to be effective. DOMS is seemingly related to muscle damage from eccentric exercise, like downhill walking, where contracting muscles are forcibly lengthened [8]. This mechanical stress triggers an inflammatory response and the production of reactive oxygen species (ROS) that sustain inflammation and oxidative stress by promoting the activation of transcription factors like the nuclear factor-κB (NF—κB), a pro-inflammatory master switch that controls the production of inflammatory markers and mediators [9]. Inflammation and oxidative stress lead to neutrophil accumulation and an increased production of the “inflammatory soup” of oxidative enzymes, cytokines and chemokines [9-11]. This eventually overcomes the antioxidant capacity of the body [12], ultimately resulting in muscle injury and DOMS. Cellular disruption is associated to direct activation and sensibilization of the transient receptor potential (TRP) ion channel family member TRPV1 via acidification and the liberation of inflammatory eicosanoids. This in turn sustains inflammation by liberation of inflammatory peptides and triggers the generation of a pain sensation (for a review, see [13]).

As a constituent of turmeric (*Curcuma longa* L.), curcumin (diferuloylmethane) has been used for centuries in the traditional medicine of India and the Far East [14,15]. Curcumin, a powerful promoter of anti-oxidant response [16], is one of the best investigated natural products [17], and is now commercially available in a lecithin delivery system (Meriva®, Indena SpA, Milan) that improves curcuminoids bio-availability. This formulation has accumulated significant clinical documentation of efficacy in various conditions triggered and/or sustained by chronic inflammation, like diabetic microangiopathy and retinopathy [18], central serous chorioretinopathy [19], benign prostatic hyperplasia [20], chemotherapy-related adverse effects in cancer patients [21] and osteoarthritis [22]. In addition, curcumin as Meriva® was also recently validated as an analgesic agent with potency at least comparable to that of acetaminophen [23].

Several studies have investigated the mechanisms by which curcumin exerts its beneficial effect. Early experimental study demonstrated that curcumin suppresses the activation of NF—κB [24,25], an effect of critical relevance in DOMS relief, since NF—κB appears to be involved in the regulation of proteolysis and inflammation in muscle [26]. Therefore, inhibition of NF—κB by curcumin may result in a muscle-protective effect. Consistently, it has been suggested that curcumin may prevent loss of muscle mass during sepsis and endotoxaemia and may stimulate muscle regeneration after traumatic injury [26,27]. Other mechanisms possibly responsible for the anti-inflammatory and anti-oxidant properties of curcumin include induction of heat-shock response [28], reduction in the expression of the pro-inflammatory enzyme cyclooxygenase-2 (COX-2) [29] and promotion of the antioxidant response by activation of the transcription factor Nrf2 [30]. Experimental evidence indicates that curcumin can reduce inflammation and decrease some of the negative effects associated with eccentric exercise-induced muscle damage, including the release of pro-inflammatory cytokines and markers of muscle injury like creatine kinase (CK) [31]. These observations provided a rationale for evaluating if curcumin, administered as a lecithin formulation (Meriva®) to improve absorption, could attenuate damage from oxidative stress and inflammation related to acute muscle injury induced by eccentric continuous exercise.

## Methods

The study was a randomised, placebo-controlled, single-centre, single-blind pilot trial. It was carried out in accordance with the Declaration of Helsinki, and was approved by the local Ethics Committee of the Consell Català de l’Esport (0099S/ 4882/2010).

The study was carried out at the Sports Physiology Dept. of the Olympic Training Center “Centre d’Alt Rendiment” of Sant Cugat del Vallés, Barcelona, Spain.

### Subjects

Twenty male healthy, moderately active (regular aerobic exercise for at least 4 hours per week), non-smoking volunteers with no known musculoskeletal pathology were recruited. Subjects had to have a maximal oxygen consumption (VO_2_max) of at least 35 ml/kg, as assessed by the maximal treadmill exercise test. Subjects were excluded if they met one or more of the following exclusion criteria: treatment with anti-inflammatory/analgesic/antioxidant drugs in the previous month, abnormal liver or renal function tests, laboratory findings suggestive of an active inflammatory or infectious process and presence of any known disease.

Proper eligibility of all subjects was evaluated by a comprehensive medical history and physical examination by a sports medicine physician.

### Supplement

Subjects were randomised (1:1) to curcumin given as the Phytosome® delivery system (Meriva®, Indena S.p.A. Milan, Italy) 1 g twice daily (corresponding to 200 mg curcumin twice a day) at breakfast and dinner, or a matching placebo. Supplementation was initiated 48 hours prior to the test and was continued for 24 hours after the test (4 days in total). Study subjects and physicians performing the radiologic and laboratory assessments were blinded to treatment, whereas the sports medicine physicians involved in exercise testing were not.

### Exercise testing

#### Maximal exercise test

Each participant completed a standardized maximal treadmill exercise test. A fixed treadmill grade (3%) was maintained throughout the test. The treadmill speed was initially set at 6 km/h, and increased by 1 km/h each minute until maximum sustainable effort (muscle fatigue or stabilisation/decline in VO_2_max) [32,33]. Maximal speed (Spd_max_), the speed at the anaerobic threshold (Spd_at_) and the VO_2_max were recorded for each participant. The tests were completed on a motorised treadmill (ERGelek EG2, Vitoria-Gasteiz, Spain). Expired air was sampled using indirect calorimetric system (Master Screen CPX, Erich Jaeger, Wurzburg, Germany).

#### Eccentric muscle injury protocol

A downhill running test modified from the protocols described by Nurenberg *et al*. [34] and Malm *et al*. [35] was used to induce the eccentric muscle injury. After a 10-min warm-up at a speed chosen by the subject, subjects ran downhill (treadmill grade -10%) at a constant speed for 45 minutes. Running speed during the 45-min exercise was to be maintained at the anaerobic threshold, which was determined prior to the test by measurement of lactate concentration in capillary blood during a 5-min run at a treadmill inclination of 3%. A speed corresponding to a lactate concentration of 3.5-5 mmol/L was considered appropriate and therefore maintained throughout the exercise protocol. Subjects performed ten-min exercise bouts during the week prior to the study day (days -7 and -5) to familiarize with the exercise protocol and to break down more susceptible muscle fibres, in order to achieve similar fibre composition and standardize the baseline level in all subject [36,37]. One hour before the eccentric injury protocol all subjects received an oral nutritional supplement containing 25 to 30 g of carbohydrates and 2–4 g of protein. Also, hydration was assured by consumption of approximately 500 mL of mineral water from 30 min. prior to the start of the test. Subjects were allowed to drink water during the test.

### Magnetic resonance imaging (MRI)

**Figure 1:**
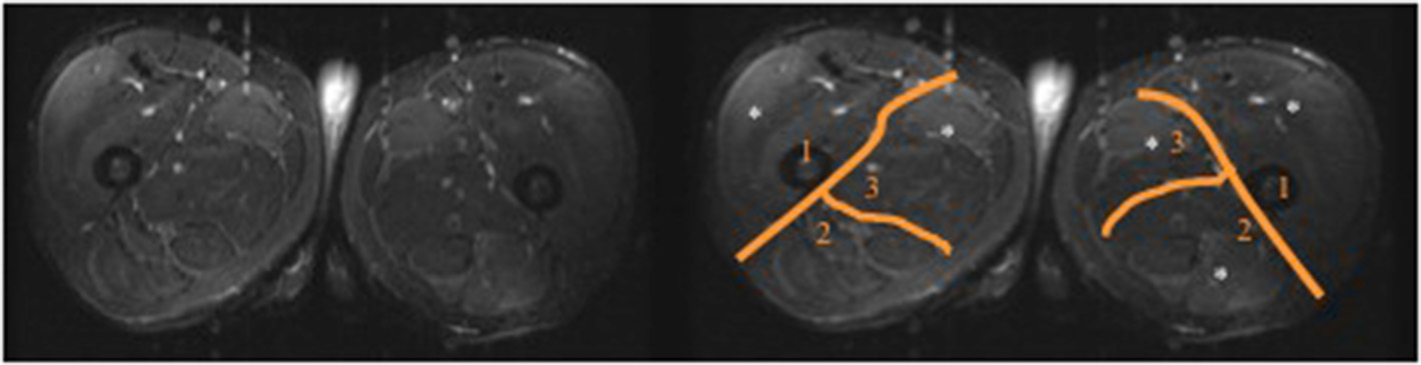
**STIR sectional image of both thighs in the middle third.** Asterisk marks muscle area with increased uptake.

A high magnetic field system was used (Signa 1.5 T, G.E. Milwaukee, WI, USA). Images were acquired 48 hours after exercise, with the subjects in the supine decubitus position. Both thighs were explored. The diagnosis was based on MRI signal alterations in any muscular group both in the flexor and the extensor compartment, as well as on signal asymmetry as compared with the contralateral homonymous muscular group. The radiologist was blinded to the treatment group. Five non-contiguous axial imaging slices (2-mm thickness, 2-mm gap) were selected. In order to quantify muscle injury, each thigh was divided into three compartments (anterior, posterior, medial) (Figure 1). A compartment was considered positive for muscular injury when an area of high signal intensity on T2-weighted and STIR sequences was observed in at least one muscle.

### Muscle biopsies

Muscle biopsies were performed 48 hours after exercise to obtain samples for the analysis of markers of cellular injury (muscle myeloperoxidase [MPO] activity, immunohistochemical analysis of albumin [38] and CD3 positive cells). A skin incision was performed with a 5 mm blade. The same skin incision was used for both muscle biopsies, changing the needle direction [34,39]*.* Two biopsies were carried out from the middle third of each *vastus lateralis*, under ultrasound control. Muscle samples were obtained using a Vacora System Biopsy gun (Bard Medical Systems, Tempe, AZ, USA), with a coaxial needle of 10G × 140 mm. With this technique the sample is aspirated and remains in the branula until it is extracted; the sample is taken out as a whole and not fragmented. Once the samples were extracted, they were kept in formol, glutaraldehyde and a third sample was cryopreserved at -80°C for further studying [40].

For histochemical procedures, all muscle specimens were first dissected free of visible connective tissue and fat and embedded in paraffin using conventional methods. Ten-micrometre sections were cut, varying the inclination of the holder by 5-degree increments until the minimum cross-sectional area was obtained, which was defined as truly transverse.

#### Evaluation of sarcolemmal disruptions

Evaluation of sarcolemmal disruption relies on the observation that cellular membrane permeability to albumin is a sign of membrane injury [41]. For assessment and quantification of muscle membrane injury, we chose a method based on light microscopy for identification of fibres that contain albumin by immunohistochemistry. Each sample was processed for immunohistochemical techniques using a polyclonal rabbit anti-human antibody directed against albumin (Code No. A0001; Dako Cytomation, DK-2600 Glostrup, Denmark) as a primary antibody. This immunocomplex was detected using a horseradish peroxidase-labelled goat anti-rabbit secondary antibody (Code No. K4003; EnVision + System-HRP labelled polymer, Dako Co., Carpinteria, CA, USA). The reaction was developed with a chromogen solution with 3.3-diaminobenzidine (Code No. K3468; Liquid DAB + Substrate-Chromogen Solution, Dako Co., Carpinteria, CA, USA). The analysis of intracellular albumin was performed by two independent observers using a categorical scale (0–3) with a light microscope (Olympus, Series AX70TF; Olympus Optical Co., Shinjukuku, Tokyo, Japan) coupled with an image-digitizing camera (View Finder Lite; Version 1.0.143c; Pixera Co., Los Gatos, CA) and a morphometry program (Scion Image, Version Beta 4.0.2; Scion Co., Frederick, MD, USA). Qualification of fibre injury was performed in a four-category finite interval system, the extremes representing either the absence of intracellular albumin (i.e., absence of sarcolemmal injury, degree 0), or presence of intracellular albumin on the complete cellular area (i.e., severe sarcolemmal injury, degree 3). The two intermediate categories were classified as degree 1 injury (i.e., mild sacolemmal injury or presence of albumin in less than 50% of the fibre area) and degree 2 injury (i.e., moderate sarcolemmal injury or presence of albumin on more than 50% of the fibre area, but not in all of it). Fibre categories were expressed as proportion (%) of total muscle fibres. The mean value of degree 2 and degree 3 obtained by two observers was used for statistical analysis [42,43].

#### CD3+ and MPO immunohistochemical staining

For the assessment and quantification of CD3+ and MPO intra/interfibrillar infiltrates we chose a method based on light microscopy for identification of CD3+ and MPO by immunohistochemistry. For the immunohistochemical assay we used manual immunostaining. For MPO a polyclonal rabbit antihuman antibody directed against MPO (Dako Ref. IS511, 1:4 dilution, prediluted), and for CD3+ a polyclonal rabbit anti-human directed against MPO (Dako Ref. A0452, 1:250 dilution), were used as a primary antibodies. For MPO antigen retrieval, the sections were deparaffinized, rehydrated in gradually decreasing concentrations of ethanol, PBS (3x5’), citrate 7,3 retrieval, pressure cooker, PBS (3x5’), hydrogen peroxide 10’ at room temperature, PBS (3x5’), primary antibody 30’ at room temperature 1:4 dilution, PBS (3x5’), secondary antibody Envison 30’ at room temperature, PBS (3x5’), DAB (1 drop for 1 ml dilute) 5/10 minutes at room temperature, PBS (3x5’), Mayer haematoxylin 10’ at room temperature, water, dehydration, D.P.X. assembly, visualization with Envison/HRP Dako (Glostrup, Denmark). For CD3+ antigen retrieval, the sections were deparaffinized, rehydrated in gradually decreasing concentrations of ethanol, PBS (3x5’), citrate 7,3 retrieval, pressure cooker, PBS (3x5’), hydrogen peroxide 10’ at room temperature, PBS (3x5’), primary antibody 30’ at room temperature 1:250 dilution, PBS (3x5’), secondary antibody Envison 30’ at room temperature, PBS (3x5’), DAB (1 drop for 1 ml dilute) 5/10 minutes at room temperature, PBS (3x5’), Mayer haematoxylin 10’ at room temperature, water, dehydration, D.P.X. assembly, visualization with Envison/HRP Dako (Glostrup, Denmark). The lymphocytic infiltrates (CD3+) and MPO were quantified following the total number of T cells immunostained antibodies against CD3+, and MPO. The total number of CD3+ cells, and the total number of fibres were counted blindly by two observers, and were used for statistical analysis. CD3+ cells per fibre was calculated and compared between PT and CT40. Number of fibres with MPO was evaluated in the same way [35,44]. The testing laboratory was blinded to treatment allocation.

### Laboratory analyses

One week prior to the study day, routine laboratory analyses (complete blood count, erythrocyte sedimentation rate [ESR], C-reactive protein [CRP], aspartate aminotransferase [AST], alanine aminotransferase [ALT], gamma glutamyl transferase [GGT], alkaline phosphatase, urea, creatinine, uric acid, total cholesterol, HDL-C, LDL-C, triglycerides, sodium, calcium, magnesium, vitamin D, serum iron, transferrin, ferritin) were performed to assess eligibility.

#### Oxidative stress and inflammatory markers

Blood samples were collected immediately before the downhill running test and 2 and 24 hours after the exercise for the measurement of CRP, high-sensitivity CRP (hsCRP), ERS, interleukin-8 (IL-8), monocyte chemoattractant protein-1 (MCP-1), ferric reducing ability of plasma (FRAP), catalase (CAT) and glutathione peroxidase (GPx). Creatine kinase (CK) was used as a marker of muscle damage.

### Pain intensity

Pain intensity was assessed 48 hours after downhill running. Patients were asked to indicate the site of pain on a drawing representing the lower limbs, and to rank pain intensity on a 0–4 point scale, where 0 = no pain and 4 = disabling pain when descending or climbing stairs. The scores relative to different sites on each side of the thigh and leg were summed to obtain a total score for each segment of the lower limb (anterior right thigh, posterior right thigh, anterior right leg, posterior right leg, anterior left thigh, posterior left thigh, anterior left leg and posterior left leg).

### Statistical analyses

Data are expressed as means ± SD [95% confidence interval]. Between- and within-group changes in IL-8, MCP-1, CK, ESR, CRP, hsCRP, FRAP, CAT and GPx levels were analysed with a two-way mixed-design analysis of variance (ANOVA) followed by Tukey-Kramer test for pairwise comparisons. Pain scores and albumin, MPO, CD3+ cells were analysed by the Hotelling’s T2 test. The Pearson’s Chi squared test was used to analyse data obtained from the MRI. Significance was set at p < 0.05.

## Results

### Study participants

Nineteen subjects out of twenty completed the study. One subject in the Meriva® group dropped out before the injury test phase by personal decision. Baseline characteristics of participants are presented in Table 1. There were no statistically significant differences between subjects in the placebo (n = 10) and the Meriva® (n = 9) group. Maximal speed reached during the maximal exercise test was 13.7 ± 1.8 [12.4;15.9] and 14.8 ± 1.1 [13.9;15.6] km/h in the placebo and Meriva® group, respectively (p = ns). During the downhill running test subjects treated with placebo and Meriva® were able to maintain a speed of 10.9 ± 1.2 [10.0;11.7] and 11.4 ± 0.9 [10.8;11.4] km/h, respectively, for 45 minutes, which was comparable to the speed at the anaerobic threshold (Table 1).

**Table 1:** Subjects’ baseline characteristics

	**Placebo (n = 10) Mean ± SD**	**95% CI**	**Curcumin (n = 9) Mean ± SD**	**95% CI**
Age (years)	38.1 ± 11.1	30.1;46.1	32.7 ± 12.3	23.1;42.1
Height (cm)	174.8 ± 3.0	172.7;176.9	176.6 ± 3.6	173.7;179.4
Weight (kg)	75.8 ± 6.5	71.2;80.4	76.2 ± 4.2	73.0;79.5
BMI (kg/m^2^)	24.8 ± 1.7	23.6;26.0	24.4 ± 1.0	23.6;25.2
VO_2_/kg (ml/kg)	45.8 ± 4.7	42.5;49.2	48.9 ± 5.3	44.8;53.1
Maximal speed (maximal exercise test) (km/h)	13.7 ± 1.8	12.4;15.0	14.8 ± 1.1	13.9:15.6
Speed at the anaerobic threshold (km/h)	10.9 ± 1.7	6.6;12.1	11.8 ± 1.5	10.6;12.9
Speed during the injury provocation test (km/h)	10.9 ± 1.2	10.0;11.7	11.4 ± 0.9	10.8;11.4

### Imaging studies

**Figure 2:**
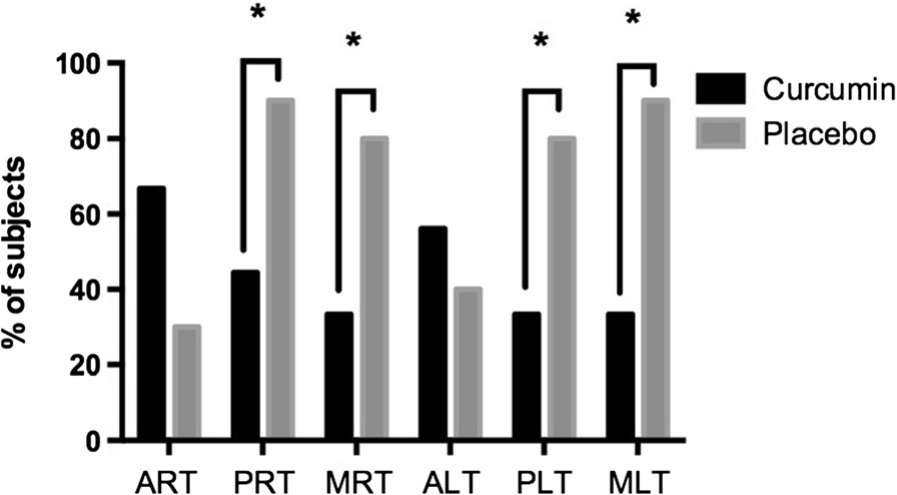
**MRI evidence of muscular injury.** Percent of subjects with MRI evidence of muscular injury. ART, anterior right thigh; PRT, posterior right thigh; MRT, medial right thigh; ALT, anterior left thigh; PLT, posterior left thigh; MLT, medial left thigh. *p < 0.05 for curcumin vs placebo.

Overall, the number of subjects with MRI evidence of muscle injury was similar in the two groups. However, the proportion of subjects with MRI evidence of muscle injury in the posterior or medial compartment of the right thigh was significantly lower in the Meriva® group as compared to the placebo group (44.4% vs. 90%, p = 0.0329 and 33.3% vs. 80%, p = 0.0397) (Figure 2). Similarly, less subjects in the Meriva® group had MRI evidence of muscle injury in the posterior or medial compartment of the left thigh (33.3% vs. 80%, p = 0.0397 and 33.3% vs. 90%, p = 0.0106) (Figure 2).

### Pain intensity

**Figure 3:**
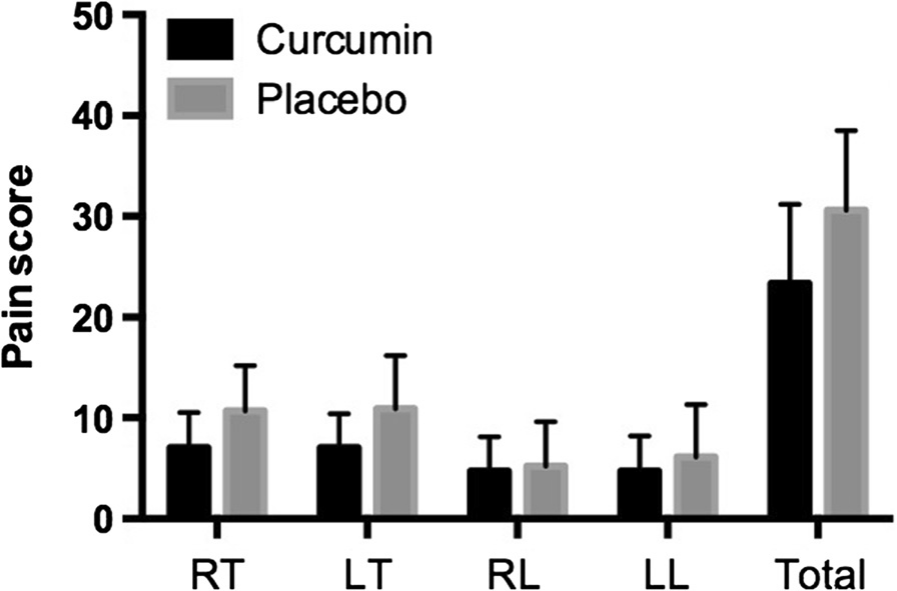
**Pain intensity.** Patient-reported pain intensity in the right thigh (RT), left thigh (LT), right leg (RL), left leg (LL) and total pain score (the sum of the scores of each lower limb).

Subjects in the curcumin group reported less pain in the lower limb as compared with subjects in the placebo group (total score: 23.3 ± 7.9 [17.2;29.4] vs. 30.6 ± 7.9 [24.9;36.2], p = 0.06) (Figure 3). However, this difference did not reach statistical significance. Similarly, the analysis of each segment considered revealed a trend for less pain in the Meriva® group, but a statistically significant difference was observed only for the right and left anterior thighs (4.4 ± 2.5 [2.6;6.3] vs. 7.8 ± 3.9 [5.0;10.6] and 4.4 ± 2.4 [2.6;6.2] vs. 8.2 ± 4.6 [4.9;11.5] in the Meriva® and placebo group, respectively; p < 0.05).

### Markers of muscle injury and inflammation

**Figure 4:**
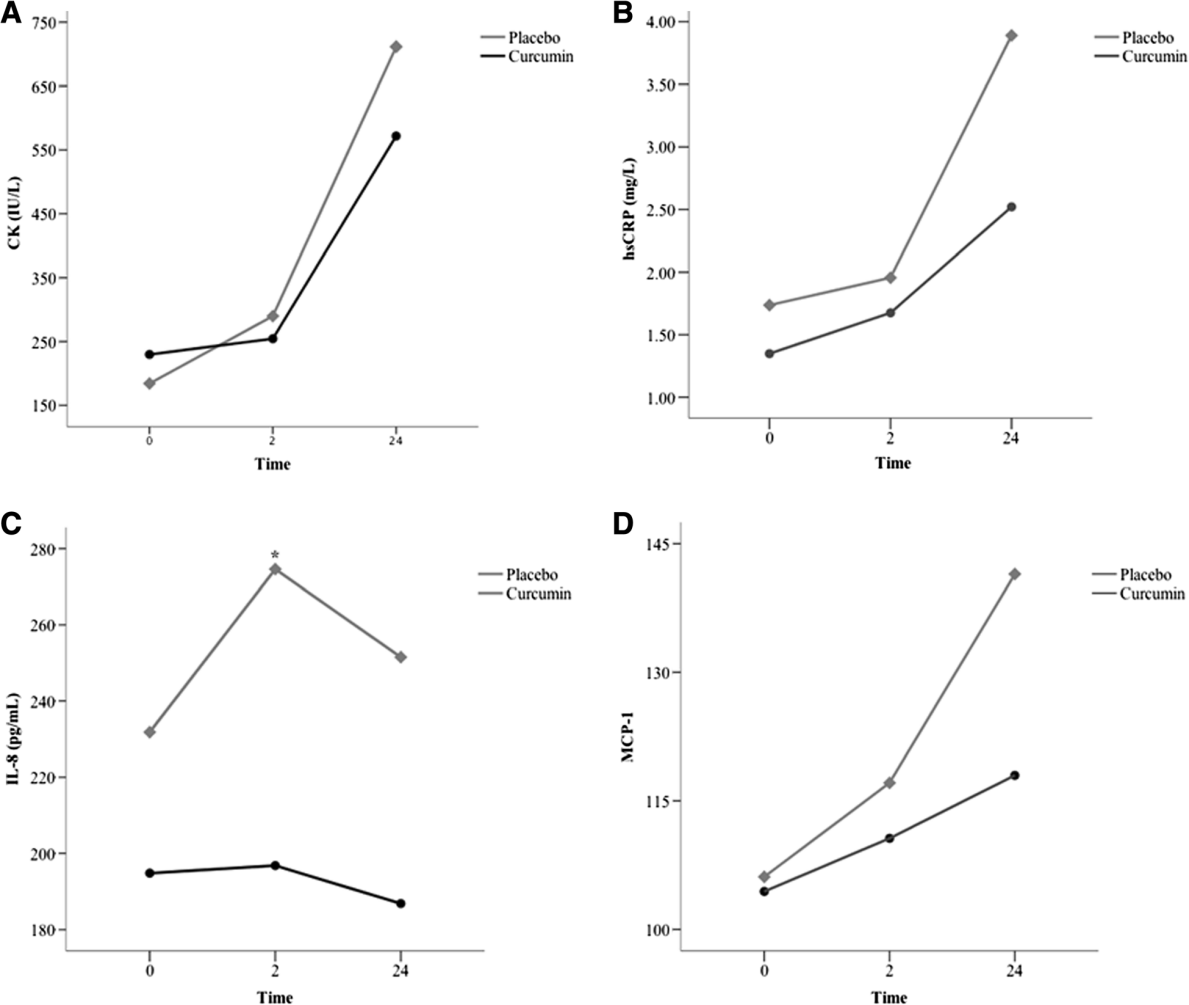
**Markers of muscle damage and inflammation. A**. Creatine kinase (CK), **B**. high-sensitivity CRP (hsCRP), **C**. interleukin-8 (IL-8) and **D**. monocyte chemoattractant protein-1 (MCP-1) levels measured at baseline and 2 and 24 hours after the downhill running test. *p < 0.05.

CK levels significantly increased from baseline in both groups, confirming the presence of muscle injury (Figure 4A). Although CK levels tended to increase less in the Meriva® group, this difference did not reach statistical significance. hsPCR levels paralleled the increase in CK, and significantly increased from baseline in both groups (Figure 4B). However, at 24 hours the percent increase from baseline was numerically lower in the Meriva® group than in the placebo group (116.2% vs. 156.1%, respectively; p = ns). IL-8 levels tended to remain stable in the Meriva® group, whereas a steep increase was observed at 2 hours in the placebo group (Figure 4C). At this time point, IL-8 was significantly lower in the Meriva® group (196.8 ± 66.1 [146.4;247.1] vs. 274.7 ± 70.7 [226.8;322.4] pg/mL, p < 0.05). No significant differences were observed in MCP-1 levels between the two groups (Figure 4D).

### Oxidative stress

Both groups experienced a modest increase in markers of oxidative stress. FRAP levels did not show significant changes over time, whereas CAT and GPx levels tended to increase at 2 hours after exercise and returned towards baseline values at 24 hours. These trends were similar in both groups.

### Muscle biopsies

**Figure 5:**
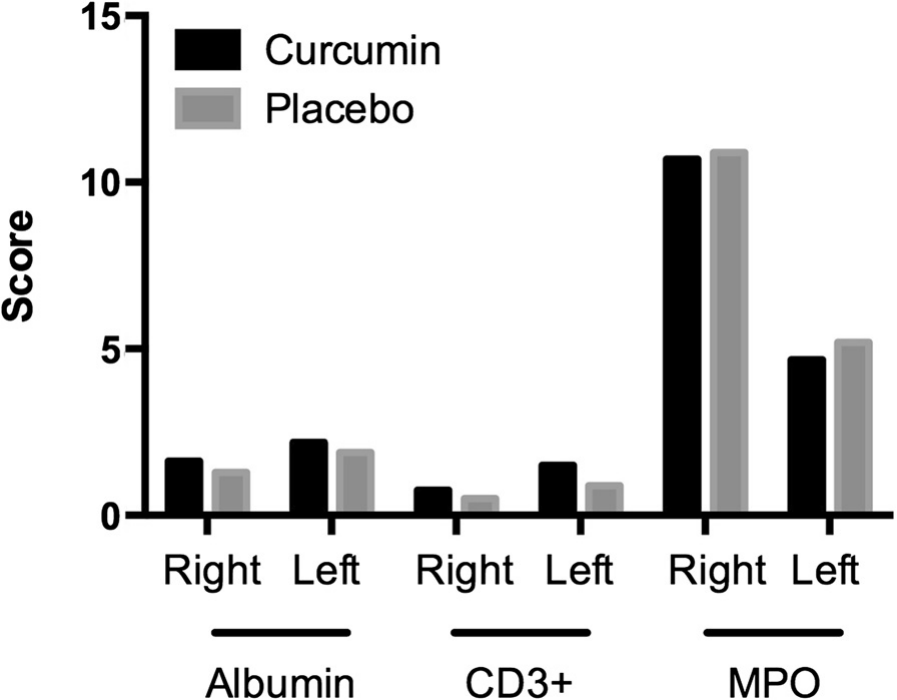
**Sarcolemmal damage and acute inflammatory response to exercise.** CD3+, CD positive cells; MPO, myeloperoxidase.

Muscle samples were available for four subjects in the curcumin group and five subjects in the placebo group. No significant differences were observed between the two groups with regard to sarcolemmal disruption and the magnitude of the acute inflammatory response to exercise (Figure 5).

## Discussion

### “Antioxidants” and exercise

In the present study we sought to investigate the effects of curcumin on damage from oxidative stress and inflammation related to acute muscle injury induced by eccentric continuous exercise. We found that curcumin supplementation reduced MRI evidence of muscle injury in the posterior or medial compartment of the thighs and was associated with a trend for less pain in the lower limb and a blunted systemic inflammatory response as compared with placebo. Several mechanisms might be responsible for the favourable effects that curcumin had on exercise-induced muscle injury in this study, but the most plausible are related to the antioxidant properties of curcumin. However, there is considerable confusion on the role of “antioxidant” supplementation and exercise. In fact, supplementation with vitamin C has been shown to decrease the development of endurance capacity [45] and the view that exercise and antioxidants might work against each other was also suggested by studies showing that anti-oxidant supplementation abrogates the beneficial effects of exercise on insulin resistance [46]. Since exercise increases consumption of oxygen and mitochondrial activity, ROS might, paradoxically, mediate not only cellular damage associated to exercise, but also its beneficial effect. Direct anti-oxidants like vitamin C and vitamin E were used in these “negative” anti-oxidant studies. These compound directly react and quench free radicals and ROS, while curcumin and phenolics are essentially boosters of the body’s endogenous antioxidant response, and exert “antioxidant” activity indirectly, by Nrf2-mediated stimulation of the cellular antioxidant system and the expression of cytoprotective genes.

### Effect of curcumin on oxidative stress and inflammation

Since curcumin can both stimulate the endogenous antioxidant response via Nrf2 activation and moderate inflammatory response via NF-kB inhibition, it could in principle be useful to increase tissue resistance to ROS while at the same time not interfering with the beneficial metabolic effects associated to their generation. In this context, it was therefore interesting to evaluate if supplementation with curcumin, administered as a Phytosome® delivery system (Meriva®) to promote absorption, could affect DOMS induced by eccentric exercise. To the best of our knowledge, this is the first study to investigate the effects of curcumin on DOMS in humans. In a previous study, curcumin supplementation was shown to improve the inflammatory pattern and markers of muscle injury, ameliorating the performance deficits associated with exercise-induced muscle damage [31]. We found that significantly less subjects in the Meriva® group had MRI evidence of muscle injury in the posterior or medial compartment of both thighs 48 hours after exercise, and a trend for lower pain intensity (p = 0.06) in the lower limb was observed in subjects receiving curcumin, with a statistically significant difference versus placebo for the right and left anterior thighs. These findings suggest that curcumin might be beneficial in the prevention of DOMS. However, one might argue that, being a mild inhibitor of cyclooxygenase 1/2 (COX1/2) [47,48], curcumin may interfere with muscle growth. In fact, the detrimental effects of non-steroidal antinflammatory drugs (NSAIDs), which are known inhibitors of COXs, are an important point of concern [49]. This effect is mediated by the inhibition of COXs, and COX2 in particular, and seems typical of all agents active on these pro-inflammatory end-points. Curcumin is a poor inhibitor of COX1/2, and its effects on the production of prostaglandins are essentially due to the inhibition of the (mPGES)-1 [50], the inducible form of the ultimate enzyme involved in the generation of the single specific prostaglandin PGE2. Inhibition of (mPGES)-1 has not been related to interference with muscle growth, that seemingly results from the global depletion of prostanoids associated to the inhibition of “uphill” enzymes involved in their generation, like COXs. Conversely, PGE2 is considered one of the markers of muscle damage induced by exercise [51].

### Analgesic effect of curcumin

In a previous study that evaluated the analgesic efficacy of the same formulation (Meriva® 2 g, corresponding to curcumin 400 mg) taken as needed in patients with acute pain, curcumin had a well-defined pain-relieving effect, even greater than that of acetaminophen 500 mg, and was better tolerated than nimesulide [23]. This acute effect is probably related to the desensitization or the inhibition of a series of transient receptor potential ion channels involved in the generation of painful *stimuli* like TRPV1 and TRPA1 [52,53]. In that study, the analgesic effect of curcumin lasted for approximately 4 hours, and a second dose, administered 6–12 hours after the first dose, was necessary for controlling pain in some cases [23]. In our study, Meriva® was administered at a dose of 1 g (delivering 200 mg curcumin) twice daily for four days, starting 48 hours prior to the exercise test and until 24 hours after exercise. The pain relieving effect of Meriva® could be mediated by a modulation of the inflammatory and oxidative responses to muscle injury. Muscle injury in DOMS appears to be related to inflammation and oxidative stress leading to neutrophil accumulation, increases in oxidative enzymes, cytokines and chemokines [9-11]. A significant increase in CK levels over 24 hours in both groups validated the protocol used in this study as an inductor of muscle damage. This increase was moderated by supplementation with Meriva®, that also led to lower levels of hsPCR and IL-8 2 hours after exercise. Several studies have confirmed that curcumin down-regulates the expression of several pro-inflammatory cytokines involved in proteolysis and muscle inflammation [25] by suppressing NF—κB signalling [54,55]. This mechanism could also be responsible for the reduced inflammatory response to exercise observed in this study.

### Study limitations

It should be acknowledged that the findings of this study may be limited to aerobic exercise, since different types of exercise (e.g., aerobic and resistance exercise) elicit unique molecular responses, and the effects of ROS in muscle may vary depending on the type of exercise involved [49]. Furthermore, markers of oxidative stress were only slightly increased after exercise in both groups, which does not allow a comparison of the effects of curcumin *versus* placebo. The failure to observe differences in tissue markers of sarcolemmal disruption and inflammatory response between the two groups of volunteers might be due the small number of muscle samples available for analysis. Previous positive studies on curcumin supplementation for chronic musculoskeletal conditions like osteoarthritis [22,56] involved longer treatments (3–8 months), and it might therefore be that supplementation in this study was too short to produce statistically significant histological benefits over placebo.

## Conclusions

Taken together, our observations suggest that curcumin may be beneficial to attenuate exercise-induced DOMS, and larger studies could provide statistical significance also for the functional and biochemical parameters that only showed a trend to improvement in our study, like the histological evaluation of muscle damage.

## Competing interests

Stefano Togni and Federico Franceschi are employees of Indena SpA, the manufacturer of Meriva®. Giovanni Appendino is a consultant to Indena SpA.

## Authors’ contributions

FD, JR, XV, AP, JT collected study data and followed patients. GA, ST, FF contributed to data interpretation and drafted the manuscript. All Authors have read and approved the final manuscript.
